# A Review of the Progress of Medical Education in Chicago, with Some Suggestions for Its Advancement

**Published:** 1881-02

**Authors:** E. Ingals


### ARTICLE V.

### A REVIEW OF THE PROGRESS OF MEDICAL EDUCATION IN CHICAGO, WITH SOME SUGGESTIONS FOR ITS ADVANCEMENT. Read before the Chicago Medical Society, Nov. 8, 1880, by E. INGALS, M.D.

My purpose in this communication is to compare the present standing of medical education in this city with the past; to determine what progress we have made, and to inquire what duties we, as a profession, owe to the future. The present is a favorable time to institute this comparison; for all that has been done in the cause of medical education here has passed under the observation and is fresh in the memory of those who are still on our streets, busy with the everyday activities of life.

I entered Rush Medical College in November, 1845, and graduated February 18, 1847. No literary attainments were then necessary to obtain admission to the class. The requirements for graduation were: A good moral character; age, twenty-one years; three years study of medicine, including two courses of lectures of sixteen weeks each; a satisfactory examination and written thesis. There were six professors. The class of 1846–47 numbered seventy matriculates, or about twelve to each professor, and sixteen, or 22.8 per cent. of the class, graduated. The dissecting room was kept open after the close of the session for such students as desired its use, and members of the faculty were always ready to give private instruction to those who would remain in the city after the lecture term. At that time there was no hospital in the city, and clinical material was scantily supplied by a dispensary in the college building, and such private patients as the professors could bring before the class. Prof. Brainard, especially, was able to present a good number of interesting cases, and such as could be obtained were fully utilized. In this way he enabled the class to see the first surgical operation which was performed in this city under anæsthesia. It was the amputation of a finger, with a portion of the metacarpal bone. Neither Dr. Brainard nor the students knew what the anæsthetic substance was. It was called letheon, and was administered by a dentist, from a bag, as the nitrous oxide gas is now given. The perfect success of the operation elicited applause from the class, that seemed electric to a degree that I have seldom witnessed in a public assembly. This will give a fair idea of the facilities for obtaining medical knowledge in Chicago in 1847, and also what was necessary to get a medical diploma here at that time—subjects that are not always closely related.

The annual announcements of the colleges of this city for the session of 1880–81 are now at hand, and an abstract of their contents should indicate what progress has been made. The Woman's Medical College requires satisfactory proof of the possession of a good moral character and of a good English educatian, for admission to the class. Applicants who have "certificates of graduation from a high school, or like institution, or a teacher's certificate from a county superintendent of schools," are received, but when these cannot be furnished, a satisfactory examination by a committee of the faculty is required. The school has thirteen professors; the class for the session of 1879–80 numbered sixty-four non-graduates, or about five to each professor; and ten—15.6 per cent.—graduated. The course of instruction is graded. Requirements for graduation are: Age, 21 years; three years study of medicine, including two courses of lectures of twenty-one weeks each, less ten days vacation during each session, and a satisfactory examination. This indicates an advance for this school of the requirement of good moral character and literary attainments for admission to the class; a lengthening of the lecture term three and a half weeks each session; an addition of seven professors—the number of pupils taught being about the same. Seven and two tenths per cent. less of the class graduated, which indicates greater care in the final examinations.

The Chicago Medical College has a graded course of instruction. Applicants, to be admitted to the class, must have graduated from some literary college, or present certificates from some scientific school or academy, or, in lieu of this, pass a satisfactory examination before a committee of the faculty. Requirements for graduation: Age, twenty-one years; good moral character; three years of medical study, including two courses of lectures of twenty-six weeks each; a satisfactory examination and written thesis. The school has fifteen professors, and the class for the session of 1879–80 numbered 148 non-graduates, or about ten to each professor; and thirty-six, or 24.3 per cent. of the class, graduated. The advance here shown is the requirement of a good English education for admission to the class, a lengthening of the lecture term ten weeks each session, an addition of nine professors—the class being about two and one ninth times larger. One and five tenths per cent. more of the class graduated, which points to less care in the final examinations.

The Rush Medical College admits all to the class who apply, without questioning their literary attainments. Requirements for graduation: Age, twenty-one years, and satisfactory evidence that the candidate possesses a good moral character, and "such primary education as is clearly requisite for a proper standing with the public and the profession;" three years of medical study, including two courses of lectures of twenty-one weeks each. The school has twelve professors, and the class for the session of 1879–80 numbered 440 non-graduates, or more than thirty-six to each professor, and 147 graduated, or 33.4 per cent. of the class. The advance shown by this school is an addition of six professors—the class being about six and one third times larger than it was thirty-three years before—a lengthening of the lecture term five weeks each session. The rule for graduation that requires "such a primary education as is clearly requisite for a proper standing with the public and the profession," looks towards an advance, but it is indefinite, and seems to stand in the wrong place. To withhold the degree from a student who has demonstrated the possession of sufficient medical knowledge to be worthy of it, because he lacks literary attainments, after he has been permitted to employ his time and incur the necessary expense of two courses of lectures, is so manifestly unjust that I feel sure the rule would never be enforced. The student should be informed of his literary deficiency when he seeks to enter the medical college, instead of when he is about to leave it. Ten and six tenths per cent. more of the class graduated, which indicates less care in the final examinations. Indeed, it is more difficult to discriminate in this particular when the applicants for graduation are many than when they are few.

All these schools have extended the curriculum of teaching; they have a greater abundance of clinical material; they have added a spring session of lectures, and though the instruction is mainly given by those who are not professors, this is a practical prolongation of the college year for such students as will avail themselves of its advantages; but to do this is not essential to graduation; and my mind has been directed in this review to the easiest means of getting a diploma in Chicago, as well as to the broadest opportunities that exist here for obtaining medical knowledge.

There are two classes of medical students: one attend lectures to get knowledge, and for these the diploma is relatively unimportant; the other think chiefly of the diploma, and will obtain it in the easiest way possible.

Many things in medicine are taught now that were not taught when I was a student, for many things are known now that were not known then, and the opportunities for obtaining medical knowledge are better than they were; and such students as will remain here and devote four or five years to mastering the text-books, hearing such lectures as profit them, taking private courses on special subjects, attending the clinics, learning anatomy in the dissecting-room and pathology in the dead-house, may qualify themselves to be competent and accomplished physicians. But the three years term of medical study which has always been required here as preliminary to an examination for the degree of Doctor of Medicine, has not been increased, and except in requiring that a larger portion of the three years should be spent in college—viz., three and a half weeks each session by one college, five by another, and ten by a third, and the requirement of a preliminary education to enter the class by two of the colleges—it is in no way more difficult to get a medical diploma in Chicago now than it was a third of a century since. In one respect it is even easier; for these colleges all belong to the Association of American Medical Colleges, and the articles of confederation of this Association provide that the degree may be conferred after but two years and nine months study of medicine. Neither do I understand how more knowledge is to be obtained now than then, except as this is influenced by the time deducted from office instruction to be added to the lecture term, though the field of study is enlarged and the subjects taught are more numerous. We could no more than master the subjects then taught, and had our field of study been enlarged, our work must have been performed in a more superficial manner. If the term of instruction is made short, the best results are reached by applying the time to fundamental branches.

While we make access to the profession as easy now as it was in 1847, in other respects the conditions of society are entirely changed. Then a laborious day of travel across the prairie would only accomplish about forty miles, and the best shelter for the night was a log cabin; now we are transported six or seven hundred miles in the same time, and take a very comfortable hotel along with us. Chicago then had less than 17,000 people; settlements were small and scattered. Everybody was poor; there was a great deal of sickness, and a scarcity of medical practitioners. These abnormal conditions had to be met by temporary expedients. The grade of medical education was placed low, but I think as high as was then wise or practicable. Now our surroundings are changed. We have a large and comparatively dense population, abundant wealth, a superfluity of medical practitioners, while improved machinery and accumulated capital give us leisure so that we can afford to those who are studying the art of healing the sick, time and money enough to enable them to perfect themselves in it to a reasonable degree before they enter upon its practice. Indeed, society cannot afford to do less than this, for the difference between the attendance on the sick of an incompetent and a competent physician, is the difference between death and life, pain and its absence, poverty and plenty. Few misfortunes impoverish more than sickness; for it both increases the consumption of wealth and checks its production; and this consideration might justify the government in prescribing the qualifications that medical practitioners should possess, and warrant it in extending aid to medical as it does to primary education.

No student should be received into a medical college who does not possess sufficient education to admit him to a literary college of respectable rank; and it would be better that he should be a graduate of such a college; for when he enters a literary college he is but commencing his literary cultivation, but when he enters the medical college, he is supposed to have completed all special literary pursuits. The mental discipline which such training gives would enable the student more perfectly to assimilate the medical instruction presented to him; his reasoning faculties would be strengthened, his powers of observation made more acute; he would become a better physician, have more influence in society, and help to exalt the rank of the profession in public esteem. It is more than a century since Blackstone wrote: "A long course of reading and study must form the physician. The gentlemen of the faculty of physic, beyond others, remarkably deserve a character of general and extensive knowledge." Can any modern Blackstone justly pay us this compliment to-day? The course of instruction should be graded. This separates the advanced student from those who are commencing the study, and students who have acquired a good deal of medical knowledge certainly require different instruction from those who have but little; it relieves the mind from the distraction incident to the pursuit of too many studies at the same time, and enables the student to get some knowledge of the fundamental branches before entering upon those that are based upon them. At least four years study, including attendance on three regular sessions of lectures of not less than eight months each, should be required before a student is admitted to an examination for graduation.

No person who has studied medicine but three years can be properly qualified to enter the chamber of the sick, to assume the responsibility of dealing with the intricate problems of disease. At the best, the physician has a large part of his professional knowledge to acquire after he has graduated, and a deficient literary and medical education leaves him unfitted to profit by his bedside observations after he has entered upon practice, and his patients have added to the perils of sickness the danger of being prescribed for by a nominal physician, who in their sick chambers is studying his profession in a blind way, without an instructor. Four years study, including three sessions of lectures of eight months each, gives one-half the period of study to college instruction, and the greater the part of the period of pupilage that is passed in the college the better.

The common experience of a student in a physician's office is that he has the use of a medical library, with some general directions of the order of his reading; but he receives little personal instruction. A physician in active practice will seldom find time or inclination for more than this. A medical college should have a large number of professors, in order to divide the curriculum of teaching into small sections, which will enable each professor more thoroughly to qualify himself to teach the subjects assigned him. There are many things in medicine that cannot be properly taught to large classes. The signs of disease revealed by a physical exploration of the body, the manipulations required in the practice of ophthalmology, otology, laryngology, gynæcology, many surgical operations, the use of instruments, the application of dressings, and the like, may be *described* to a large class, but they cannot be *taught* to a large class. This is as impossible as it would be to teach the art of making horse-shoes by didactic lectures on the craft of the blacksmith. Yet no one is fitted to enter upon general practice who has not received careful instruction on most of these subjects. Another benefit of a subdivision of the class is that this brings each of its members into nearer personal relations to his teacher, which is often a great advantage aside from the medical instruction received, for the professor should be a model on which the student may mold his character, and principles thus imparted may favorably influence his entire subsequent life.

The examinations for the degree should be rigid, and if there is doubt of the competency of the applicant, he should be rejected. This is the more necessary as the principal evidence usually required as proof that the student has attended two courses of lectures, is that he holds the tickets for two courses of lectures. He may absent himself from the lectures, or he may take out the tickets near the close of the session, and yet be admitted to the examinations for graduation the same as if he had heard all the lectures. Sometimes, too, faculties are imposed upon by false certificates of study; but care in the examinations would detect these frauds. The percentage of those examined for the degree who are rejected is so small, that it serves as a practical invitation to all who have complied with certain easy formalities, to apply for it. The medical diploma should be conclusive evidence that its possessor is a learned person, but, as conferred by many schools, it cannot be accepted as sufficient proof.

At a largely attended session of the annual meeting of the Illinois State Medical Society, for the year 1879, the following resolutions were offered:

"*Resolved*, 1st, that the Illinois State Medical Society requests of all regular medical colleges that they institute preliminary examinations for students who apply for admission to their classes, and only admit such as have, at least, a thorough English education.

"*Resolved*, 2d, that the annual sessions of lectures by the regular faculties should not be of shorter duration than six months.

"*Resolved*, 3d, that all students should be required to study medicine five years, and attend three full annual sessions of lectures, before they are admitted to examination for the degree of Doctor of Medicine."

The first two resolutions were adopted unanimously, and the last with but few negative votes.

At the annual meeting of the alumni of the Rush Medical College for 1879, the following was ordered to be printed in the transactions, but action on it was postponed until the next annual meeting, the purpose of the postponement being that the subject might be well considered before action on it was taken:

"The Alumni of Rush Medical College desire to express, in a formal and public manner, the affectionate regard we entertain for our Alma Mater, our pride in her past history, and confident hopes for her increased usefulness in the future. We hold that her supreme duty to the public, which has always accorded her a generous support, demands that her first aim and object should be to aid in exalting the attainments of the medical profession, and by so doing to increase its usefulness, power and respectability. As a mode of accomplishing these results, we would respectfully offer to the board of trustees of the college the following suggestions:

*First.* To increase the regular term of college instruction to a period of not less than nine months. *Second.* To require attendance on three full terms of medical lectures as a prerequisite for admission to examination for the degree of Doctor of Medicine. *Third.* To institute preliminary examinations for students who apply for matriculation to the school, and admit only such as have, at least, a thorough English education."

I judge that 200 of the alumni were present at the annual meeting of 1880, when these resolutions were adopted without a dissenting voice. The action of these organizations, as above expressed, is doubtless in harmony with the individual opinions of most of the members of the profession. The medical education of physicians in Illinois is mainly left to the profession, and a faithful performance of the work is a duty we owe to society. This duty does not rest alone on our medical schools, but is shared with them by the entire profession, and it is this which causes me to bring the subject before this society for its consider-tion. The review I have made does not show that the institutions of medical learning in this city have assumed the advanced position the profession seems to demand. Though something has been accomplished, it is less than we had hoped, and to my mind, the promise of the immediate future is not encouraging.

The last announcement of Rush Medical College contains this notice: "On and after March 1, 1883, all applicants for admission to the college will be examined as follows: In the elements of physical science as taught in the common school text-books; in arithmetic to cube root." Is it possible that this rule is looked upon as an advanced requirement? and if so, why is its operation postponed three years? Is so much time necessary to enable medical students to prepare for the ordeal of an examination that is limited by the text-books of our common schools? and do we expect our profession to be accorded a place among those recognized as learned, while we confer its honors and impose its duties on persons whose literary attainments are so low that they are unable to "cipher to cube root"? Is this a sufficient preparation for those whose daily avocations require a knowledge of such subtle and intricate subjects as chemistry, histology, physiology, pathology, whose prescriptions are to be written in a classical language, and who are to uphold the character of our profession in their associations with cultivated people?

I wish it was as easy to make the grade of the profession higher as it is to demonstrate that it is now too low. I have no definite plan of how this purpose may be effected, but will offer some suggestions that I hope you may supplement by others. As individual members of the profession, and associated in our medical societies, we should continue the agitation of the subject. We should bring every influence we can to bear on those who have the immediate conduct and control of our medical schools.

We should educate the profession and the public up to a higher standard. Some schools have already been influenced in their action on this subject by an enlightened wisdom, and they merit our highest commendation.

But whatever we may accomplish with the regular schools, we can have little hope that our influence will affect those that we do not recognize, and these commission practitioners whose incompetency people learn through suffering and death. Such schools make fiat doctors as it is proposed to make fiat money, by the printing press, and each are of about equal value for the purposes for which doctors and money were instituted. The action of the schools in this matter is perhaps restrained by the fear that an increase of requirements would lessen the number of students, and consequently the amount received from fees, and give to the schools an appearance of diminished prosperity.

But the schools should learn that their standing will be fixed, not by the number, but by the acquirements of their graduates. If the requirements were much raised, the number annually graduated would certainly be less; but if by this means the number could be reduced one-half, it would be a great gain, not only to the profession, but to society, and also to those who should be thus turned away from an employment for which they are not fitted by their attainments. But neither the aggregate of the fees nor the numbers in the classes would be so much reduced, for the students would remain longer under instruction. But even if the professors' fees should be reduced, we must keep in mind that medical schools ought to be conducted in the interests of students and of the general public, rather than in the exclusive interests of the limited number of persons who give instruction in them. The latter course would be like establishing common schools for the pecuniary and other advantages they might afford to the teachers, instead of for the purpose of educating children and benefiting society. The object in view could perhaps be promoted by legislation. For example, laws might be enacted that after some time in the not distant future, no person who had not then been admitted to practice in this State, should be allowed to commence the practice here unless he was a graduate of a medical college that required four years of medical study, three annual sessions of medical lectures of not less than eight months each, laboratory work, practical anatomy, and hospital instruction, as a prerequisite for graduation, and the possession of a good English education for admission to the class. It would be well, too, if legal provision could be made for a State examining board, whose duty it should be to examine all applicants for medical graduation in the State, so that an equal thoroughness of teaching should be exacted of all our schools. These examinations might include materia medica, but exclude therapeutics, which would avoid all questions growing out of special systems of practice. The colleges should confer the degree on the recommendation of this board.

If the suggestions I have made are not attainable, then a good influence on the cause of medical education might be exerted by establishing a new medical college in Chicago, on a basis similar to what is recommended above. Should this be thought best, such an enterprise is practicable. Other advantages than those growing out of increased requirements for graduation might result from establishing another school in this city, for the classes that annually assemble here, even now, are too large to be as well taught as they could be if they were smaller; and as our population increases—other things remaining the same—the number of students will also increase. Chicago is as well suited to become a great medical center as it is to become a great manufacturing and commercial center. By its railroad ramifications it will be easily accessible to a large and wealthy population; its climate is favorable for health and study; it will afford abundant material for clinics and dissection; the necessary cost of living here will be small; the city will contain numerous objects of interest to invite students to it; and these factors will cause it to be the residence of many skillful practitioners and able teachers. We should lay the foundations here as we would that those who come after us should build.

